# Comprehensive Analysis of the NHX Gene Family and Its Regulation Under Salt and Drought Stress in Quinoa (*Chenopodium quinoa* Willd.)

**DOI:** 10.3390/genes16010070

**Published:** 2025-01-09

**Authors:** Yalla Santhoshi, Asha Bindhu Anjana, Harshvardhan Zala, Tejas Bosamia, Kapil Tiwari, Ketan Prajapati, Pranay Patel, Nishit Soni, Nitin Patel, Satyanarayan Solanki, Ulhas Sopanrao Kadam

**Affiliations:** 1 Department of Genetics and Plant Breeding, C. P. College of Agriculture, Sardarkrushinagar Dantiwada Agricultural University, Sardarkrushinagar 385 506, Gujarat, India; 2 Plant Omics Division, CSIR-Central Salt and Marine Chemicals Research Institute (CSIR-CSMCRI), Bhavnagar 364 002, Gujarat, India; 3 Bio-Science Research Centre, Sardarkrushinagar Dantiwada Agricultural University, Sardarkrushinagar 385 506, Gujarat, India; 4 Division of Applied Life Science (BK21 Four), Plant Molecular Biology and Biotechnology Research Center, Gyeongsang National University, Jinju 52828, Republic of Korea

**Keywords:** antiporter, ion transporter, NHX gene, abiotic stress, gene expression level, salinity stress, drought stress

## Abstract

**Background/Objectives**: Abiotic stresses such as salinity and drought significantly constrain crop cultivation and affect productivity. Quinoa (*Chenopodium quinoa* Willd.), a facultative halophyte, exhibits remarkable tolerance to drought and salinity stresses, making it a valued model for understanding stress adaptation mechanisms. The objective of this study was to identify and characterize Sodium/Hydrogen antiporter (NHX) genes from the quinoa genome and study their role in stress tolerance. **Methods**: We identified and characterized 10 NHX genes from the quinoa genome, which belong to the monovalent cation/proton antiporter 1 (CPA1) superfamily. Comprehensive analysis, including phylogenetic relationships, motif patterns, and structural characteristics, was performed to classify these genes into three subfamilies. Physicochemical properties such as isoelectric point (pI), GRAVY, and transmembrane domains were examined. Promoter analysis was conducted to identify *cis*-elements linked to abiotic stress responses, phytohormone signalling, and light regulation. qPCR analysis was used to assess the differential expression patterns of *Cq*NHX genes under salt and drought stress. **Results**: The analysis revealed that the NHX genes were divided into three subfamilies localized to vacuolar, plasma, and endosomal membranes. These genes exhibited structural and functional diversity. Promoter analysis indicated the presence of *cis*-elements associated with abiotic stress responses, phytohormone signalling, and light regulation, suggesting diverse regulatory roles. qPCR analysis revealed differential expression patterns of *CqNHX* genes under salt and drought stress, with vacuolar NHXs showing higher induction in leaf tissues under salinity. This underscores their critical role in sodium sequestration and ion homeostasis. Evolutionary analysis indicated a high degree of conservation within subfamilies, alongside evidence of purifying selection. **Conclusions**: The findings enhance our understanding of the molecular basis of stress tolerance in quinoa and provide valuable targets for genetic engineering to improve crop resilience to environmental challenges.

## 1. Introduction

As sessile organisms, plants must endure abiotic stresses such as drought, salinity, and extreme temperatures. Abiotic stresses are one of the significant constraints for crop cultivation and productivity. Salinity impairs plant growth in two phases: initial osmotic stress hampers water uptake and nutrient absorption, leading to reduced growth. The next phase involves building up of ion toxicity that disrupts metabolic processes, accelerates aging, and triggers oxidative stress [1]. Drought stress in soil is a significant challenge in arid and semi-arid regions caused by rising temperatures. Drought impacts 45% of the world’s agricultural land [2]. Water stress mainly causes stomatal closure, which decreases relative water content (RWC) and the rate of photosynthesis [3,4].

Quinoa (*C. quinoa* Willd.), an allotetraploid crop originating from South America [5], is highly nutritious, containing significant amounts of protein, carbohydrates, essential amino acids, vitamins, minerals, and antioxidants [6,7], and is recognized globally for its exceptional nutritional qualities and abiotic stress tolerance. Peru and Bolivia dominate global quinoa production, accounting for 97% in 2020. While cultivation in India is gaining attention, it covers only 440 hectares, producing 1053 tonnes [8]. The crop is considered a salt- and drought-tolerant plant and categorized as a facultative halophyte, which may withstand salt concentrations of 150 to over 750 mM NaCl [9,10]. Harnessing genomic resources from the stress-tolerant crop quinoa has attracted significant interest from researchers. Several stress-responsive genes have been identified and characterized for their roles in mitigating biotic and abiotic stresses, such as Superoxide dismutase [11], Glutathione S-transferase [12], SNF1-Related protein kinases Type 2 [13], High-affinity K^+^ transporter [14], Salt Overly Sensitive [15], Protein phosphatase 2C [16], Pyrabactin resistance 1 like [17], and β-amylase [18].

The Na^+^/H^+^ antiporter (NHX) genes belong to the monovalent Cation/H^+^ transporters (CPA1) family, which is part of the large cation-proton antiporters (CPA) superfamily [19]. These proteins function as pumps and are found within cell and organelle membranes. They possess a distinct Na^+^/H^+^ exchanger domain, which plays a crucial role during stress by transporting Na^+^ ions in exchange for H^+^. Most NHX transporters typically maintain 10–12 transmembrane domains and include a potential amiloride-binding site (FFIYLLPPI), where amiloride binds with exceptionally high affinity. Amiloride functions as a competitive inhibitor for Na^+^ ions by obstructing their binding sites [20]. Based on the subcellular localization of the transporter, they are classified into three groups: vacuolar, plasma-membrane, and endosomal localized NHX proteins [21]. The vacuolar-bound NHX proteins help compartmentalize Na^+^ ions to prevent their toxicity inside the cell [22]. NHX1 and NHX2 in Arabidopsis control growth, flowering, reproduction, and pH and K^+^ concentration in vacuoles [23]. NHX1, an endosomal member of the NHE gene family in yeast, controls vesicle trafficking by regulating luminal and cytoplasmic pH [24]. In grapevine, *VvNHX1* also plays a crucial role in development and adaptation, influencing seed dormancy, growth, ripening, and stress responses [25]. Seven *PtNHX* genes from *Populus tomentosa* [26] and nine *CsNHXs* from tea [27] are responsive to multiple stresses such as salt, drought, cold, heat, methyl jasmonate, and abscisic acid (ABA) treatment. Arabidopsis lines expressing *GmNHX3* and *GmNHX1* genes have been analyzed in transgenic soybean [28]. The NHX gene family has been studied in numerous plant species such as maize [29], wheat [30], rice [31], sugar beet [32], mulberry [33], alfalfa [34], cotton [35], soybean [36], rapeseed [37], tomato [38], chickpea [39], cucurbits [40], and honeysuckle [41] under salt stress. Subsequently, the significance of NHX genes under drought stress has been demonstrated in several plants [42,43,44,45,46,47,48,49]. However, the structural and functional characterization of quinoa’s NHX genes remains unstudied.

The present research aimed to identify and characterize NHX genes in quinoa to elucidate their potential role in enhancing salt and drought stress. In total, 10 NHX genes were retrieved from quinoa. By integrating various in silico analyses like evolutionary relationship, conserved motifs, multiple sequence alignment, gene structure, pair-wise similarity, chromosomal mapping, gene duplications, and promoter profiling with qRT-PCR expression profiling, we sought to uncover the molecular mechanisms underlying NHX gene function in quinoa.

## 2. Material and Methods

### 2.1. Identification of NHX Genes in Chenopodium Quinoa

The genome and protein databases of *Arabidopsis thaliana* and *C. quinoa* were sourced from the Phytozome (https://phytozome-next.jgi.doe.gov/ (assessed on 14 January 2024)). To identify potential *CqNHX* genes in *C. quinoa*, we used eight AtNHX sequences as queries for local BLAST alignments. The NHX-like domain sequences were filtered using TBtools v1.108 [50]. The NCBI-Conserved Domain Database (NCBI-CCD) (https://www.ncbi.nlm.nih.gov/cdd (accessed on 14 January 2024)) (Marchler-Bauer and Bryant, 2004) and Simple Modular Architecture Research Tool (SMART) (http://smart.embl-heidelberg.de (accessed on 14 January 2024)) [51] were used to validate the retrieved protein sequences for the NHX domain.

### 2.2. Physiochemical Properties and Subcellular Location Prediction

The sequences of all the CqNHX proteins were imported into the Plant-MPLoc server [52] to predict their subcellular location. The different physiochemical parameters such as isoelectric point (pI), molecular weight (MW), instability index, aliphatic index, exon/intron ratio, and Grand average of hydropathicity (GRAVY) of the NHX proteins were obtained using the online tool ExPASy ProtParam (https://web.expasy.org/protparam/ (accessed on 15 January 2024)) [53]. Transmembrane helices (TMHs) of the NHX proteins were estimated by using TMHMM server v2.0 (https://services.healthtech.dtu.dk/services/TMHMM-2.0/ (accessed on 15 January 2024)) [54]. The SignalP v6.1 server (https://services.healthtech.dtu.dk/services/SignalP-6.0/ (accessed on 15 January 2024)) [55] and Netphos 3.1 (https://services.healthtech.dtu.dk/service.php?NetPhos-3.1 (accessed on 15 January 2024)) [56] web tool were used for the prediction of signal peptide information and phosphorylation sites of the CqNHX proteins, respectively.

### 2.3. Protein Sequence Alignment and Phylogenetic Analysis

The NHX protein sequences of rice, maize, Arabidopsis, sorghum, and wheat were obtained from the literature [30,41]. The multiple sequence alignment was performed using the ClustalW, version 2.0 application, followed by converting the resultant file into MEGA format. The phylogenetic analysis was performed using MEGA11 (https://www.megasoftware.net/history (accessed on 16 January 2024), PHP) [57]. Furthermore, a circular evolutionary tree was created through a neighbor-end joining approach with 1000 bootstrap replicates and visualized using iTOL v6 [58].

### 2.4. Conserved Motif and Intron/Exon Structure Analysis

The protein sequences of 10 CqNHXs were scanned to identify the conserved motifs using the Multiple Expectation Maximization for Motif Elicitation program (MEME version 5.5.5, http://meme-suite.org/tools/meme (accessed on 19 January 2024)) [59]. The working parameters were defined as follows: the base width could vary from 6 to 50 amino acids, and the maximum number of motifs allowed was 10. The CDS sequences of each of the 10 NHX genes were compared with their respective genomic DNA sequences using Gene Structure Display Server 2.0 (GSDS, http://gsds.cbi.pku.edu.cn/ (accessed on 19 January 2024)) [60] to predict the exon/intron distribution pattern in the CqNHX genes.

### 2.5. Prediction of Chromosomal Localization, Gene Divergence, and Cis-Acting Elements

The *C. quinoa* genome database was obtained from the CoGe (https://genomevolution.org/coge/ (accessed on 4 February 2024)) web server using Genome id-33827 [61] and was utilized to know the positions of 10 NHX genes on each chromosome. The datum was then employed to generate a genetic map with the help of Map Gene 2 Chromosome (MG2C v2) (http://mg2c.iask.in/mg2c_v2.0/ (accessed on 5 February 2024)) [62] using default parameters. TBtool-II was used to analyze the synonymous (Ks) and non-synonymous (Ka) substitution rates of duplicated CqNHX gene pairs by adapting the Nei and Gojobori method [63]. The resultant Ka/Ks ratio was used to determine the selection mode.

The promoter sequences of 1500 bp upstream of quinoa NHX genes were retrieved from the Phytozome database (http://www.phytozome.net (accessed on 20 January 2024)). Subsequently, these promoter sequences were utilized to predict the cis-acting regulatory elements using the PlantCARE website (http://bioinformatics.psb.ugent.be/webtools/plantcare/html/ (accessed on 20 January 2024)) [64].

### 2.6. Growth Conditions of Plant Material and Stress Treatments

In this study, quinoa seeds of the genotype Him Shakti were obtained from the Center of Crop Improvement (CCI), S. D. Agricultural University, Sardarkrushinagar. The seeds were sown in the greenhouse at the Bioscience Research Centre and Botanical Garden, Department of Genetics and Plant Breeding, C. P. College of Agriculture, S. D. Agricultural University, Sardarkrushinagar, in January 2023. Him Shakti is a new, locally developed variety that has been specifically adapted to Indian conditions. The variety possesses superior nutritional value, with a high protein content of 15.64% and oil content of 8.91%. It also demonstrates enhanced resilience to various stresses, making it ideal for breeding programs.

Seeds of uniform size and fullness, free of diseases and pests, were sorted, sterilized in 10% sodium hypochlorite for 15 min, washed five times with sterile water, and then planted in pots with a 1:1 mixture of sandy loam and cocopeat. Approximately 10–15 seeds were sown per pot, then transferred to the greenhouse and maintained at 20–25 °C with natural light and regular watering. Once the seedlings reached the 3–4 leaf stage, they were thinned to 6–7 seedlings per pot. After 4 weeks, the plants were subjected to stress treatments: (1) salt treatment with 300 mM NaCl, with quinoa leaves and roots sampled at 0, 6, 12, and 24 h post-treatment [65], and (2) drought treatment by water ceasing, with samples collected at 0 (control), 3, 5, and 7 days after watering stopped [13]. Three biological replicates were performed for each treatment. Samples were flash-frozen in liquid nitrogen and stored at −80 °C.

### 2.7. Differential Expression Analysis Using Quantitative Real-Time PCR

A 100 mg sample of each replicate (root and leaf samples) was collected for the total RNA extraction. RNA extraction was performed with the help of an RNeasy^®^ Plant Mini Kit (Qiagen, Hilden, Germany) following the manufacturer’s recommendations. A RevertAid First Strand cDNA Synthesis Kit (Thermo Fisher Scientific, Waltham, MA, USA) was used to synthesize first-strand complementary DNA (cDNA). The resultant cDNA was subsequently diluted at a 1:5 ratio with nuclease-free water, followed by taking 1 µL of the diluted cDNA as a template for real-time PCR analysis. A Thermo Scientific Maxima SYBR Green qPCR Master Mix (2X) kit was used to quantify the transcript levels of quinoa NHX genes in the 7500 Real-Time PCR System (Applied Biosystems, Foster City, CA, USA) according to the manufacturer’s instructions. Gene-specific primers were designed using Primer3Plus (https://www.bioinformatics.nl/cgi-bin/primer3plus/primer3plus.cgi (accessed on 27 March 2024)), and their details are listed in Supplementary Table S1. To normalize the expression of CqNHXs, the CqTUB-9 gene was employed as an endogenous control. The experiment was conducted under the following conditions: 2 min at 50 °C (UDG pre-treatment), 10 min at 95 °C (Initial denaturation), followed by 40 cycles of 15 s at 95 °C (Denaturation), 30 s at 60 °C (Annealing), and 30 s at 72 °C (Extension). The melt curve analysis was performed at 95 °C for 15 s, 60 °C for 1 min, and 95 °C for 30 min. To ensure the reproducibility of the data, reactions were set up in triplicate for each sample. The fold transcript change of *CqNHX* genes was calculated using the 2^−ΔΔCT^ method where ΔΔCT = {(CT gene of interest − CT housekeeping gene) treatment − (CT gene of interest − CT housekeeping gene) control} [66].

## 3. Results

### 3.1. Identification and Phylogenetic Analysis of the CqNHX Gene Family

The eight AtNHX protein sequences from *Arabidopsis* were searched for CqNHX proteins in the *C. quinoa* database, and 10 complete NHX peptides were revealed (Supplementary Table S2). Domain analysis using NCBI-CDD and SMART revealed that all the discovered proteins were NHXs. Subsequently, the physio-chemical properties including number of exons, strand orientation, length (amino acids), molecular weight (MW), isoelectric point (pI), GRAVY (grand average of hydropathicity), aliphatic index, instability index, subcellular localization, and transmembrane helices of predicted CqNHX proteins were ascertained through comprehensive analysis, and the results are presented in Supplementary Table S3. The number of exons in CqNHX proteins varied from 14 to 23 exons. The length (in terms of the number of amino acid residues) of the CqNHX proteins was variable, with the smallest peptide (AUR62005112-RA) consisting of 510 amino acids and the most prominent peptide (AUR62003491-RA) having 1211 amino acids. AUR62005112-RA (56.304 kDa) and AUR62003491-RA (133.817 kDa) were the smallest and largest peptides, respectively, in terms of molecular weight (kDa), which followed the same pattern as the length. Among the 10 CqNHXs identified, five were predicted to be oriented in a 5′-3′ (+) direction and the remaining five were noticed to be positioned in a 3′-5′ (−) direction. The predicted isoelectric points (pI) of the CqNHXs observed that the majority of the proteins were acidic, with pI less than seven, except AUR62015923-RA (9.18), AUR62024750-RA (8.06), and AUR62005112-RA (8.97). Given the positive GRAVY value, all the proteins were hydrophobic, with the lowest and highest values shown by AUR62003491-RA (0.088) and AUR62005112-RA (0.586), respectively. All proteins had aliphatic indices ranging from 100.62 to 114.86, indicating higher thermostability. The number of transmembrane helical domains (TMs) of the NHX proteins ranged from 9 to 12. The length of TMs was in the range of 213.81–246.16 amino acids. None of the CqNHXs were secretory proteins since none had any signal peptide in their structure. The subcellular localization prediction by the Plant-mPLoc server revealed that two NHX proteins, AUR62017800-RA and AUR62003491-RA, were localized in the plasma membrane while the rest were positioned in vacuoles.

The NetPhos tool identified potential phosphorylation sites in the 10 CqNHX proteins, revealing that UNSP, PKA, PKC, and CDC2 might exhibit the highest levels of kinase activity (Supplementary Table S4). Protein Kinase C (PKC) and PKA phosphorylation sites were noted as characteristic features of transporters [67]. Other kinases, such as INSR and SRC, were predicted to phosphorylate only a subset of the NHX proteins.

**Figure 1:**
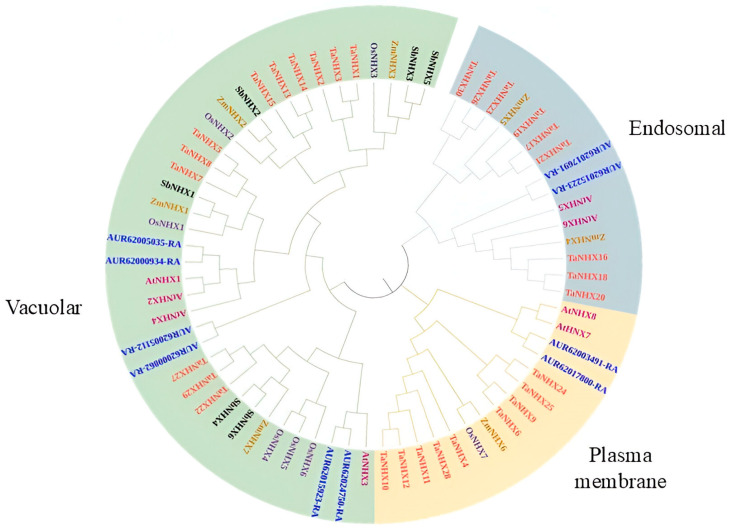
Phylogenetic relationship of NHX proteins from *C. quinoa*, *A. thaliana* (AtNHXs), *Oryza sativa* subsp. *Indica* (OsNHXs), *Triticum aestivum* (TaNHXs), *Sorghum bicolor* (SbNHXs), and *Zea mays* (ZmNHXs). The tree was constructed using the Neighbour-end joining method with 1000 bootstrap replicates.

The evolutionary relationship of the CqNHX genes across different species (Figure 1) was constructed using protein sequences of quinoa (10 *CqNHXs*), Japonica rice (seven OsNHXs), Arabidopsis (eight AtNHXs), sorghum (six SbNHXs), wheat (30 TaNHXs) and maize (seven ZmNHXs), adapting the Neighbor-Joining (NJ) method. The NHX proteins were divided into plasma membrane (PM), vacuolar (Vac), and endosomal (Endo) subfamilies based on AtNHX protein categorization. The majority of the NHXs were categorized under the vacuolar subfamily, which comprised 6 CqNHXs, 6 OsNHXs, 4 AtNHXs, 4 ZmNHXs, 12 TaNHXs, and 6 SbNHXs. Both PM and Endo subfamilies had an equal distribution of 15 NHXs each. The two CqNHXs, 2 AtNHXs, 1 OsNHX, 1 ZmNHX, and 9 TaNHXs were positioned under the plasma membrane subfamily. The endosomal subfamily contains 2 CqNHXs, 2 AtNHXs, 2 ZmNHXs, and 9 TaNHXs.

### 3.2. Prediction of Conserved Motif, Pairwise Similarity, and Gene Structure of CqNHX Genes

**Figure 2:**
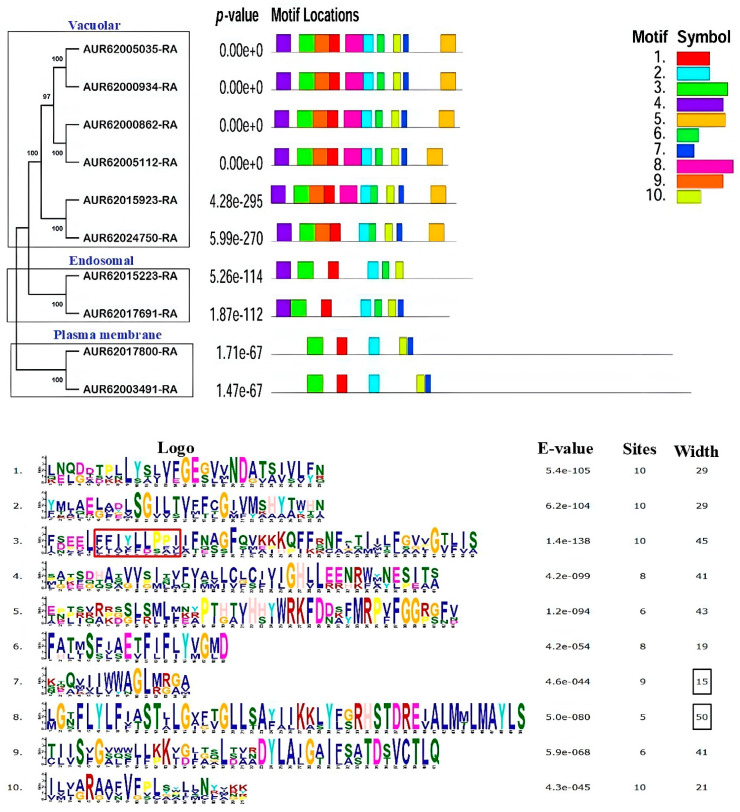
Phylogenetic relation of the NHX proteins in quinoa and their conserved motif distribution. Proteins belonging to the same subfamily are enclosed in black boxes. The identified motifs are indicated by different colors. The sequence logos refer to 10 conserved motifs of CqNHXs. The conserved region “FFIYLLPPI” is represented by motif 3 (Red Box). The width of each motif (expressed in terms of amino acids) is given in the right-hand column. The height of the letters indicates the degree of conservation at that position. Less expectation (E) value explains higher confidence of prediction. The motifs are arranged according to their E value (low to high).

Based on the evolutionary relationships among the identified CqNHXs, motif patterns, and their gene architectures, the 10 NHXs were categorized under three subfamilies. The proteins AUR62005035-RA, AUR62000934-RA, AUR62000862-RA, AUR62005112-RA, AUR62015923-RA, and AUR62024750-RA were placed under the vacuolar subfamily. The plasma membrane subfamily consisted of AUR62017800-RA and AUR62003491-RA and the endosomal subfamily of AUR62015223-RA and AUR62017691-RA (Figure 2). The MEME 5.5.0 software examined the motif distribution to understand the structure-based classification of the CqNHXs, predicting 10 putative motifs with amino-acid lengths ranging from 6 to 50 (Figure 2).

**Figure 3:**
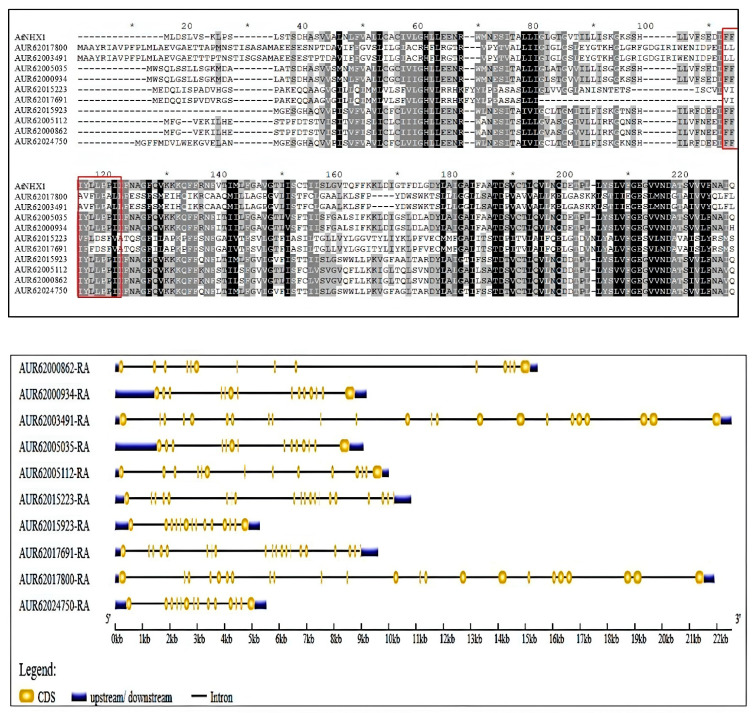
Multiple sequence alignment of amiloride-binding site (FFIYLLPPI) between quinoa NHXs and AtNHX1. The conserved sequence is separated by a box (red color). The region was present only in Vac-typed NHXs at their N-terminus. Exon and intron organization (gene structure) of *CqNHXs* was predicted by the GSDS webserver. The CDS (coding sequence) is represented by the color yellow, UTRs (untranslated regions) are shown by the color blue, and introns are indicated with solid black lines. The scale of gene lengths is given in kilo bases (kb) at the bottom.

A comparable motif arrangement was seen in the proteins that belonged to the same category. Of the 10 motifs, 2, 3, and 10 were present in all the 10 NHXs of quinoa. All 10 motifs were predicted in almost all Vac NHXs except AUR62024750-RA, which was devoid of motif 8. Motifs 4 and 6 were located in both the Vac and PM subfamilies. Motifs 9 and 5 were particular to the Vac subfamily, providing evidence for the potential loss of protein sequences during evolution. Also, the proteins under the PM subfamily showed five identical motif numbers. The Endo NHXs AUR62015223-RA and AUR62017691-RA displayed six and seven motifs, respectively. The analysis also showed that motif 8 was the largest, having 50 amino acids, and motif 7 was the smallest, having only 15 amino acids. Additionally, motif 3 was observed to have a conserved region, “FFIYLLPPI,” which was present in all 10 NHX proteins of quinoa. Amiloride, an inhibitor of NHXs, has a stronger affinity for this region. The distribution of this conserved site was again analyzed through multiple sequence alignments performed using the GeneDoc web tool. Here, the sequence “FFIYLLPPI” was presented by the Vac NHXs only (Figure 3). Also, the location of the amiloride binding site was observed to be relatively conserved among these six NHXs with its positioning at the N-terminus. To learn more about the evolutionary history of the CqNHXs, their gene structure and exon distributions were identified. Exon/intron structural heterogeneity, an essential component of gene family evolution, adds more proof to support phylogenetic classifications. The number of exons and introns in the structures of genes from the same lineage was comparable. The upstream and downstream regions were noticeably present in all the 10 CqNHXs. The PM-localized NHX genes exhibited more exons (23), followed by the Endo NHXs with 20 exons. Meanwhile, the Vac genes show a comparatively smaller number of exons (14) (Figure 3).

The EMBOSS Needle was used to calculate amino acid sequences’ identity and similarity percentages to evaluate the conservation among the CqNHX proteins. The analysis revealed that the overall amino acid sequence similarity and identity of the different CqNHXs ranged from 17.1% to 99.1% (Table 1) and 10.2% to 98% (Table 2), respectively. Higher sequence similarity and identity were shown by two CqNHX paralogous pairs in the Vac sub-class, viz., AUR62000934-RA/AUR62005035-RA = 99.1% and 98%; AUR62005112-RA/AUR62000862-RA = 93.6% and 92.7%, followed by one paralogous pair of the PM sub-class (AUR62003491-RA/AUR62017800-RA = 93.1% and 92.7%), respectively. However, the proteins belonging to different sub-classes exhibited less similarity and identity, varying between 17.1% (AUR62017691-RA/AUR62003491-RA) to 45% and 10.2% (AUR62024750-RA/AUR62017800-RA and AUR62017691-RA/AUR62003491-RA) to 24.8% (AUR62005112-RA/AUR62017691-RA), respectively. It was also observed that the similarity and identity percentages of the proteins under the PM and Endo subfamilies with their other member were 93.1%/92.7% and 84.2%/82.5%, respectively, while the different members of the Vac subfamily showed a wide range of similarity and identity percentages, beginning with 59.3% (AUR62024750-RA/AUR62000934-RA) and 46.8% (AUR62024750-RA/AUR62000934-RA and AUR62024750-RA/AUR62000862-RA) and rising to 99.1 % and 98% (AUR62 000934-RA/AUR62005035-RA).

### 3.3. Chromosome Location, Gene Divergence, and Promoter Analysis

**Figure 4:**
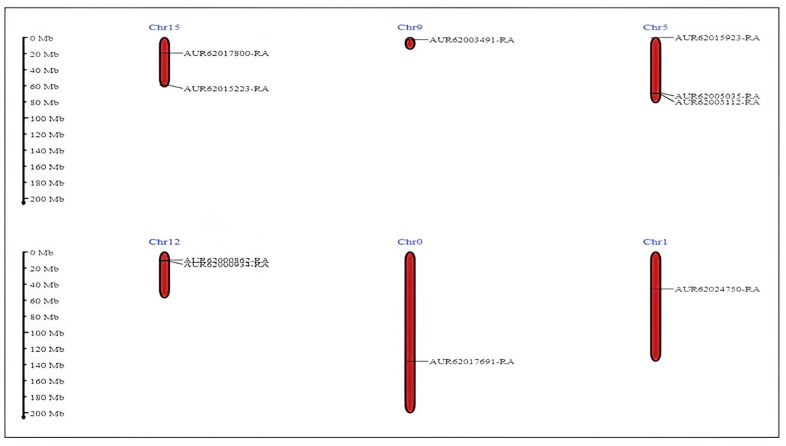
Chromosomal distribution of *CqNHX* genes. The chromosome number is shown at the top and the position of NHX genes is indicated using solid black lines. On the right of each chromosome, the gene name is labelled. The scale of genome size is indicated in terms of mega bases (Mb) on the left. Ch0—unassembled chromosome.

The chromosomal locations of the CqNHX genes were analyzed (Figure 4). The 10 CqNHXs were unevenly distributed across 6 of 18 quinoa chromosomes. The divergence of the CqNHX genes was analyzed using the non-synonymous/synonymous (Ka/Ks) ratio, with all predicted gene pairs showing a Ka/Ks ratio below 1, indicating purifying selection (Supplementary Table S5). Duplications were categorized as either segmental (gene pairs on different chromosomes) or tandem (gene pairs on the same chromosome) based on their chromosomal positions; among 23 duplications, only 3 were tandem, while the majority were segmental.

**Figure 5:**
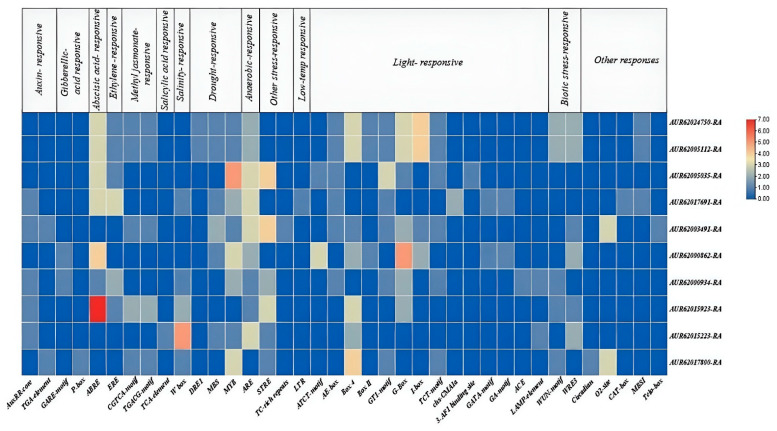
Representation of cis-acting regulatory elements present in 1500 bp upstream of *CqNHX* promoters. The functional distribution of each cis-acting elements is presented at the top. The scale of abundance is shown on the right-hand side, with a dark red color and dark blue color indicating the higher and lower abundance of cis-acting elements, respectively.

Using the PlantCARE web tool, 38 cis-regulatory elements (CAREs) regulating the 10 quinoa NHXs were identified (Figure 5). These elements were categorized into five functional groups: (1) phytohormone-responsive (auxin, gibberellic acid, abscisic acid, ethylene, methyl jasmonate, and salicylic acid), (2) abiotic stress-responsive (salt, drought, anaerobic induction, low temperature, and others), (3) light-responsive, (4) biotic stress-responsive, and (5) others involved in cellular functioning and development. Of the 38 CAREs, 9 were associated with hormone responses, with ABRE (ABA-responsive element) being the most common, found in 7 CqNHXs, followed by the ethylene-responsive element (ERE). The salt-responsive W-box element was identified in 5 NHXs. Notably, the drought-responsive MYB element and the anaerobic-responsive ARE element were found in 9 NHXs each. Promoter analysis also revealed a high abundance of light-responsive elements in the CqNHXs.

### 3.4. Expression Profiling of CqNHX Genes Under Salt and Drought Stress

**Figure 6:**
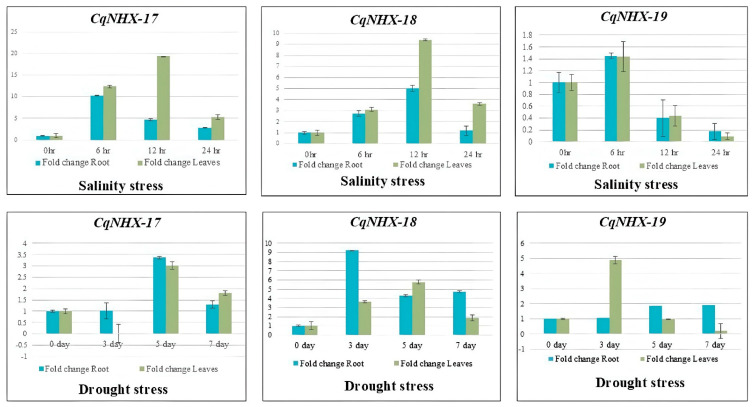
Relative expression levels of *CqNHX* genes in leaves and roots of *Chenopodium quinoa* in response to salt stress for 0, 6, 12, and 24 h and drought stress for 0, 3, 5, and 7 days. The *y*-axis and *x*-axis represent the fold change and treatment periods, respectively. Error bars indicate standard error of CT means of NHX genes.

The time course expression profiling (0 h, 6 h, 12 h, and 24 h) of three CqNHX genes in root and leaf tissues during salt and drought stress is shown in Figure 6. Under salt stress, the expression levels of AUR62000934-RA (CqNHX-17), a Vac member, showed significant upregulation, with peak expression at 12 h (~19.4-fold) and 6 h (~10.3-fold) in leaf and root, respectively, while the expression was decreased to ~5.26-fold in leaf and ~2.79-fold in root tissues at 24 h. Similarly, another vacuolar NHX, AUR62015923-RA (CqNHX-18), was induced significantly throughout the treatment, with the highest-fold change of ~9.42 in the leaf and ~5.02 in the root at 12 h. However, AUR62015223-RA (CqNHX-19), an endosomal member, showed minimal upregulation at 6 h with ~1.43-fold change in leaf and ~1.45-fold change in root, whereas its expression after 12 and 24 h of treatment was significantly downregulated in comparison with the control in both root and leaf. In response to drought stress, the expression of AUR62000934-RA (CqNHX-17) in roots was not altered after 3 days (~1.02) but significantly increased at 5 days (~3.36) and maintained a lower level at 7 days (~1.28). In leaf tissues, transcripts of AUR62000934-RA (CqNHX-17) were initially suppressed at 3 days after drought treatment compared with the control but showed significant upregulation at 5 days (~3.02 fold) and gradual decrement at 7 days to ~1.79-fold. AUR62015923-RA (CqNHX-18) showed high transcription during stress periods compared to the normal conditions (control). In root, the expression was increased to ~9.24-fold after 3 days, ~4.26-fold after 5 days, and ~4.74-fold after 7 days of drought treatment. The leaf exhibited fold change of ~3.60 (3 days), ~5.77 (5 days), and ~1.87 (7 days), which was higher than the control. AUR62015223-RA (CqNHX-19) showed slight upregulation in root, with maximum expression at 7 days (~1.91-fold). However, in leaf tissues, the gene exhibited sharp upregulation at 3 days (~4.89), thereafter gradually downregulating to ~0.98 (5 days) and ~0.19 (7 days).

## 4. Discussion

The present investigation revealed the existence of 10 NHXs in quinoa, similar to *P. granatum* [20]. However, there were 7 NHX genes discovered in *S. lycopersicum* [38], 8 in *C. arietinum* [39], 5 in *B. vulgaris* [32], 30 in *T. aestivum* [30], and 9 in *G. max* [36]. This variance in NHX gene counts among plant species suggests that events involving gene duplications or losses happened during the evolution of the species [41].

The physio-chemical analysis revealed significant diversity among quinoa NHX proteins despite similarities in amino acid length, transmembrane domains, and localization. Notable variations in properties like pI, GRAVY, and aliphatic index highlight the complexity of NHX antiporters. Similar variations in physio-chemical properties were observed in SbNHXs [68] and BvNHXs [32]. The subcellular location of NHX antiporters has a significant impact on their functionality. These CqNHX proteins were predicted to be localized in plasma membranes and vacuoles. A similar arrangement was found in NHXs from tomatoes [38] and rice [31].

The phylogenetic tree divided the 10 CqNHX proteins into three subfamilies, namely plasma membrane (PM), vacuolar (Vac), and endosomal (Endo). There were six CqNHXs in the Vac subfamily, two in the PM subfamily, and the Endo subfamily comprised two. Comparable results have been reported in wheat [30], cotton [35], and cucurbits [40]. Large numbers of vacuolar NHXs explain Na^+^ compartmentalization as a primary response in quinoa under salt stress. According to the designed phylogenetic tree, it was perceived that CqNHX transporters are not only involved in maintaining Na+ ion homeostasis but can also regulate endosomal pH and vesicle trafficking. However, these results were inconsistent with the sub-cellular localization predicted by Plant-mPLoc. The motif analysis disclosed that the Vac NHXs, except AUR62024750-RA, contain all the identified motifs. Also, the proteins under the PM subfamily showed five identical motif numbers. However, the Endo NHXs AUR62015223-RA and AUR62017691-RA displayed six and seven motifs, respectively. Considering the motif distribution, it was interpreted that PM NHXs are highly conserved compared to Vac and Endo proteins. Similarly, the NHXs from cucurbits showed a unique motif pattern, with some motifs shared by proteins that belonged to the same sub-class. Motifs 7 and 12 were exclusive to the Vac subfamily, while motif 13 was found only in the PM and Endo subfamilies [40]. Multiple sequence alignment performed using the GeneDoc webtool predicted that the sequence “FFIYLLPPI” would be present only in Vac NHXs at their N-terminus, and these results showed deviation from those predicted by MEME. All the 10 NHXs of quinoa exhibited the Na^+^/H^+^ domain of different sizes. These differences may affect the protein’s overall structure and functionality. Domain length can impact binding characteristics, stability, and interactions [69]. These results agree with those for cucurbits [40] and rice [31]. The results of gene structure represented the existence of 14, 23, and 20 exons in Vac, PM, and Endo NHXs, respectively. Since exons are the coding part of a gene, their conserved nature may explain the functional similarity of the genes that belonged to the same subfamily. These findings suggest that the families of NHX genes in different plant species exhibit structural diversification. Despite significant differences in protein length among NHX family members, the exon numbers remain conserved across various sub-classes. This shows that members with close evolutionary relationships have similar biological functions.

The analysis of pairwise sequence similarity revealed that the NHX proteins from the same subfamily displayed a higher percentage similarity of 55.3% to 99.1% and identity of 46.8% to 98%. In contrast, those belonging to different sub-classes exhibited less similarity, and identity varied between 17.1% to 45% and 10.2% to 24.8%, respectively. The findings also indicate more sequence conservation among the PM and Endo subfamilies themselves than among the Vac subfamily. These results are concordant with the findings of [41], who reported that the percentage identity among amino acid sequences of different LjNHXs varied from 8.7% to 78.9%, and the corresponding sequence similarity ranged from 13.8% to 83.6%. Also, it was stated that the proteins LjNHX2 and LjNHX5, which are members of the Vac subfamily, displayed a greater sequence identity of 78.9%. Gene duplications often occur in plant genomes due to a variety of processes, such as whole-genome duplications (WGDs), segmental duplications, and local duplications. The numerous copies of the same gene produced by these duplications inside the genome could be referred to as a gene family. Gene families are vital in evolutionary research as they offer insights into gene diversification and functional specialization [70]. The gene divergence was assessed through the Ka/Ks substitution ratio, which revealed that purifying selection is the common selection type of CqNHXs. Similar results were reported in SlNHXs [38] and CaNHXs [39].

Promoter profiling revealed that light-responsive elements exhibit most quinoa NHX regulation. EREs and ABREs in the promoter region of CqNHXs indicate their influence on ethylene and ABA signaling pathways, respectively. Also, the presence of the drought-responsive element MYB and the anaerobic-responsive element ARE indicates that the genes may participate in drought and anaerobic stress responses. These findings suggest the possible involvement of these transporters of quinoa in crucial plant regulatory events such as stress adaptations, phytohormonal signaling, and cellular NHX development. This knowledge expands on our understanding of the regulatory mechanisms governing the participation of the CqNHX gene family in physiological processes in quinoa. The overall distribution of cis-elements was comprehended to be random and unique among the 10 NHXs of quinoa, and similar results have been observed in sorghum [68] and Japonica rice [31]. Both the SvNHXs and OsNHXs also showed regulatory elements related to adaptation to stress and hormone signal response.

To further understand the possible functions of the CqNHX genes in response to salt and drought stress, the expression levels of three CqNHXs were investigated through quantitative real-time PCR analysis. The overall expression levels of Vac-class NHX genes in the leaf were significantly higher than in the root under salt stress, implying that the leaf tonoplast Na^+^/H^+^ antiporters might play a crucial role in the adaptation of quinoa in a saline environment through the sequestration of excessive Na^+^ into vacuoles of leaves. These results are comparable with the finding of [32], where under high salt treatment of 200 and 300 mM NaCl, the transcription levels of vacuolar BvNHX1 and BvNHX3 were much higher in the leaves of sugar beet than in the roots. Similarly, the salt-induced differential expression profiles in the NHXs were previously reported in *Populus trichocarpa* [26] and *S. bicolor* [68]. The NHX genes of quinoa under drought stress exhibited distinct expressional patterns, suggesting they may facilitate coordinated and temporal responses based on the duration and severity of drought stress. However, the overall induction of CqNHXs was noticed to be higher under salt compared to drought. The significance of NHXs in providing resistance under drought stress was previously investigated through expression analysis by [42], who evidenced that the 25 StNHX genes responded uniquely under 15% PEG6000 treatment and most of them were upregulated in order to provide tolerance to drought stress. The findings suggest that by stimulating diverse molecular and physiological reactions, NHX genes may improve the plant’s resistance to various environmental stressors.

## 5. Conclusions

The comprehensive analysis of the CqNHX gene family in *C. quinoa* offers valuable insights into their structural and functional characteristics, evolutionary relationships, and stress-responsive roles. A total of 10 NHX genes were identified that are scattered randomly on six chromosomes. They were classified into vacuolar, plasma membrane, and endosomal subfamilies based on their phylogenetic relationships and motif distributions. The presence of salinity and drought-responsive cis-elements in NHX genes provides evidence for their role in abiotic stress tolerance. The qRT-PCR results revealed that the gene *CqNHX-18* was intensively upregulated throughout salt and drought stress periods. Nevertheless, upregulation of *CqNHX* genes was more pronounced under salinity conditions compared to drought stress. The current study, while lacking insight into NHX gene interaction with other stress pathways, provides a foundation for further functional analysis. These findings again offer potential avenues for genetic engineering and genome editing to improve quinoa’s resilience to adverse conditions.

## Figures and Tables

**Table 1:** Pairwise sequence similarity of CqNHX proteins expressed in percentage.

Protein	1	2	3	4	5	6	7	8	9	10
1	100.0									
2	93.1	100.0								
3	20.0	19.8	100.0							
4	20.5	19.6	99.1	100.0						
5	18.9	18.3	40.2	39.9	100.0					
6	17.7	17.1	42.4	41.2	84.2	100.0				
7	18.0	18.3	64.3	64.5	38.7	40.1	100.0			
8	18.6	19.2	62.0	59.3	39.7	38.3	81.8	100.0		
9	20.8	19.9	73.9	73.7	40.2	41.1	64.5	62.0	100.0	
10	18.9	18.2	71.8	71.6	43.4	45.0	64.2	62.1	93.6	100.0

**Table 2:** Pairwise sequence identity of CqNHX proteins expressed in percentage.

Protein	1	2	3	4	5	6	7	8	9	10
1	100.0									
2	92.7	100.0								
3	10.8	11.2	100.0							
4	10.8	11.1	98.0	100.0						
5	10.9	10.3	23.3	23.6	100.0					
6	10.5	10.2	24.5	23.6	82.5	100.0				
7	10.4	10.3	52.0	51.8	23.6	24.7	100.0			
8	10.2	10.6	49.2	46.8	24.4	24.0	80.6	100.0		
9	12.4	12.2	57.6	56.9	23.4	23.5	49.0	46.8	100.0	
10	11.9	11.1	56.1	55.6	24.7	24.8	49.6	47.1	92.7	100.0

## Data Availability

The original contributions presented in this study are included in the article/Supplementary Material. Further inquiries can be directed to the corresponding authors.

## Supplementary Materials

The following supporting information can be downloaded at https://www.mdpi.com/article/10.3390/genes16010070/s1. Table S1: The sequence of primers validated using qRT-PCR; Table S2: Sequence information of CqNHXs gene, protein, and their regulators retrieved from *Chenopodium quinoa* genome; Table S3: Physiochemical properties of NHX genes from *Chenopodium quinoa*; Table S4: The phosphorylation sites identified in *CqNHXs*; Table S5: Ka/Ks substitution ratio, duplication, and selection types of NHX genes in *Chenopodium quinoa*.
