# Supplementary material for: Comprehensive Analysis of the NHX Gene Family and Its Regulation Under Salt and Drought Stress in Quinoa (Chenopodium quinoa Willd.)
Source: Genes (Basel). 2025 Jan 9;16(1):70. doi: 10.3390/genes16010070 (PMC11765057; doi:10.3390/genes16010070)
Supplement: Supplementary file 1 [file genes-16-00070-s001.zip › Table S1.pdf]

Table S1: The sequence of primers validated using qRT-PCR

Sr. No.	Protein Name	Primer Name	Primer sequence	Length	GC%	Tm	Amplicon size
1	Alpha-Tubulin 9	*TUB9-F*	GAGATGTTCCGTCGTGTGAGTGAG	25	52.0	60.0	168
		TUB9-R	ATCGGCAGTTGCATCCTGGTATTG	24	50.0		
2	AUR62000934-RA	*CqNHX-17F*	GGCGTGGGTGAACAAAGTAG	20	55.0	60.5	233
		CqNHX-17R	AGCAGAATCACAACCCCAGT	20	50.0	59.6	
3	AUR62015923-RA	*CqNHX-18F*	CTGGTGGCTGCTTCCTAAAG	20	55.0	60.0	157
		CqNHX-18R	CCCTTCCCCAAAAACTAAGC	20	50.0	59.9	
4	AUR62015223-RA	*CqNHX-19F*	TGTTGACCAATACTCCCCAAA	21	42.9	60.2	203
		CqNHX-19R	AACTCCTGCTGCTTGTTGCT	20	50.0	60.2	
