# Supplementary material for: Comprehensive Analysis of the NHX Gene Family and Its Regulation Under Salt and Drought Stress in Quinoa (Chenopodium quinoa Willd.)
Source: Genes (Basel). 2025 Jan 9;16(1):70. doi: 10.3390/genes16010070 (PMC11765057; doi:10.3390/genes16010070)
Supplement: Supplementary file 1 [file genes-16-00070-s001.zip › Table S2.pdf]

Table S2: Sequence information of CqNHXs gene, protein, and their regulators retrieved from Chenopodium quinoa genome

Gene ID	Protein ID	Sequence
CqNHX-11	AUR62024750-RA	MGFFMDVLWEKGVELANMGESGHAQVVPISVFVAVLCFCLVIGHLLEENRWLNESITAIVIGCLTGMIILFISKGKNSHILRFDEELFFIYLLPPIIFNAGFQVKKKQFFQNFLTIMLFGVIGVFISTTIISLGSWWLLPKVGFAGLTARDYLAIGTIFSSTDTVCTLQVLNQDDTPLLYSLVFGEGVVNDATSVVLFNAIQKLNVKKVGDWTHVLGSFLYLFFTSTALGIGTGLLTAYALKALYFGRSCVASFRLLQLAELSGILTVFFCGIVMSHYAWHIFATMSFIAETFIFLYVGMDVFDMEKWKMTKLSIWTLLGIYGTIIFLILLARAAFVFPLSALSNRMNREKEISGRRSSITFKKQVIIWWAGLMRGAVSIALAFKQFTYSGVTMDTVNATMVTTTVIVVLFTTLVFGFLTKPLIFYLVPHSPTQEPKSSKEDLTLPMLSMDESTATNLSRAKDSLSMLFERPIHTIHSYWRKFDDAYMRPFFGGPSFDEPALRGEVSCGYFALSGETVWGGYMREDMIRRQLQP
CqNHX-12	AUR62005112-RA	MFGVEKILHESTPFDTSTVISITVFISLICLCIIIGHLLEENRWANESITSLLLGVASGGIVLLIRKGQNSRILVFNEELFFIYLLPPIIFNAGFQVKKKQFFKNFSTILSFGVVGTLISFCLVSVGVQFLLKKIGLTQLSVNDYLALGAILSATDSVCTLQVLNQDDTPLLYSVVFGEGVVNDATSIVLFNAVQSLDLSNLSAMTALALLGTFLYLFVTSTLLGVFVGLLSAFIIKKLYLSRHSTDREIALMMLMAYLSYMIAELMDLSGILTVFFCGVVMSHYTWHNVSESSRITSKHAFATMSFISETFIFLYVGMDTLDIDKWKESNASLGTYLAVSSAMLSLILIGRAAFVFPLANILNYTKKNSDKKINFSTQFIIWWAGLMRGAVTVALSYNAFSGSVEVSNERSLMISCTLIVVLFSTLLFGTVTKPLIRAILGAPRHAVSYAKPRQRRGGFSLLMKYPTGAVHHIWRKFDNKFMRPVFGGRGFVTYEPASPTGAADISHEQ
CqNHX-13	AUR62005035-RA	MWSQLSSLLSGKMDALATSDHASVVSMNMFVALLCGCIVIGHLLEENRWMNESITALLIGLATGVVILLISGGKSSHLLVFSEDLFFIYLLPPIIFNAGFQVKKKQFFRNFITIILFGAVGTLVSFTIISFGALSIFKKLDIGSLDLADYLAIGAIFAATDSVCTLQVLNQDETPLLYSLVFGEGVVNDATSVVLFNAIQSFDLTTIDHRIALQFMGNFLYLFIASTILGAFTGLLSAYIIKKLYFGRHSTDREVALMMLMAYLSYMLAELFYLSGILTVFFCGIVMSHYTWHNVTESSRVTTKHAFATLSFVAEVFLFLYVGMDALDIEKWRFVSDSPGTSVAVSSILLGLIMVGRAAFVFPLSWLMNFAKKSHSEKVSLNQQVVIWWAGLMRGAVSMALAYNQFTRSGHTQLRGNAIMITSTITVVLFSTMVFGLLTKPLIMFLLPQPKHFTSCSTVSDLGSPKAYSPLLEGHQDYEVVGNHDNGTEPTIVRPSSLRMLLNAPTHTVHHYWRKFDDSFMRPVFGGRGFVPFVPGSPTEQSTHNLADRT

CqNHX-14	AUR62017691-RA	MEDQQISPVDVRGSPAKEQQAAGVGILLQIMMLVLSFVLGHVLRRHKFYYLPEAS ASLLIVIIFFDSFVATQSGFILAPKPFFSNFGAIVTFSVLGTFIASILTGLLVYLGGITYLI YKLPFVECMMFGALITSTDPITVLAIFQELGTDVNLYALVFGESVLNDAVAISLYRS MSALKGQSSDQNVFTFFLRFIETFAGSMSSVFTLVNIGLYLIYCLGGYILCDLDCMN LFKYAGLDIENLQNLECCLFVLFPYFSYMLAEGLGMSGIVSILFTGIVMKHYTYSNL SEKSQQFVSGFFHLISSLAETFIFIYMGLDIVMEKHSWSHIGFIFFSIITIVLARAVNVF SCGYLVNLVRPAHRKIPMTHQKALWYNGLRGAMAFALALQSVHDLPEGHGQTIFT ATTAIVVLTVLLIGGTTGTMLESLQVIGDTTEGHLDGFNRNDGFVSSGQEDEGSSSN RFKMRLKEFHRSAPSFSSLDKNYLTPFFTTQTGDEEEEENLMPTPRREVYSTRN
CqNHX-15	AUR62003491-RA	MAAYRIAVPFPLMLAEVGAETTTPTNSTISGSAMAEESESTPTDAVIFFGVSLILGIA CRHFLRGTRVPYTVALLIIGIGLGSLEYGTKHGLGRIGDGIRIWENIDPELLLAVFLPA LLFESSFSMEIHQIKRCAAQMILLAGPGVLISTFCLGAALKLSFPYDWSWKTSLLLG GLLSATDPVAVVALLKELGASKKLSTIIEGESLMNDGTAIVVYQLFLKMILGRTFNW ASILKYLVQVTFGAVGFGIAFGIASVLWLGFIFNDTVIEITLTLAVSYVAYFTAQEGA DVSGVLTVMTLGMFYAAAARTAFKGESQQSLHHFWEMVAYIANTLIFILSGAVIAQ GVLSSDNIFENHGNASVSKLVSNYMSMEDVNGLGIEKWIQHHTLGRLSDLSVGVV LFELDFDGTAWGYLILLYVYVLVARGVVVGVLYPFLCYFGYGMEWKEAMILVWA GLRGAVALSLSLSVKRSSGDPAYLSTQTGTLFVFFTGGIVFLTLIINGSTTQFVLRFLG MDKLSKAKRRILEFTKYEMEKKALEAFGDLGEDEELGPADWPTVKRYIKSLNTISG DRIHPHDASDTSDNGFLDPMNLKDMRVRLLNGVQSAYWVMLDEGRITQSTANVL MQSVDEALDAVDHEPLCDWKGLKNSVHFPKYYRLLQGGIYPKKLVTFFTVERLES ACYICAAFLRAHRTARGQLHDFIGDSEISSAVITESETEGEEARKFLEDVRTTFPEVL RVVKTRQVTYAVLQHLIEYIESLEKAGILEEKEMLHLHDAVQTDLKRLVRNPPTVKI PKIGELISMHPFLGALPSGVRDLLVGSTKEEVKVRGMTLYKEGGKPNGIWLISNGV VKWASKVRKNKHALHQTFTHGSTLGLYEVLIGKPYLCDMITDSVAVCFYIETEKIL AALGSDPAVEHFFWKESVIVLAKVLLPRVFENMSMQDMRKLTAERSTLNTYLRGE TIEVPSHSIGFLLEGFIKSHSLVEELITSPAALWPAQGNSSFLSQEGSGYKSTSFLHQG ASYYVETRARVLLIDMVPIQADNTLLRRKSSLLLHDQSSRSLNSRDHAGLLSWPEN QYKSHQRLPDGQEIGDSQNLSAKAMRLSIYGSTARDVPLRGLSFQGYSLGNPSHVR SYPQVPIGQKQRPLTSVKSEGSNTVRKRLGEDVMREELLPQTHSRHPSRVVDDSSS ESGGEDEVIVRIDSPSKLSFRQAP
CqNHX-16	AUR62000862-RA	MFGVEKILHESTPFDTSTVISITVFISLICLCIIIGHLLEENRWANESITSLLLGVASGG VVLIRKGQNSRILVFNEELFFIYLLPPIIFNAGFQVKKKQFFKNFSTILSFGVVGTLISF

		CLVSVVQFLLKKIGLTQLSVNDYLALGAILSATDSVCTLQVLNQDDTPLLYSVVFG EGVVNDATSIVLFNAVQSLDLSNLSAMTALALLGTFLYLFITSTLLGVLVGLLSAFII KKLYLSRHSTDREIALMMLMAYLSYMIAELMDLSGILTVFFCGVVMSHYTWHNVS ESSRITSKHAFATMSFISETFIFLYVGMDTLDIDKWKESNASVGTYLAVSSAMLSLIL IGRAAFVFPLANILNYTKKNSDKKINFSTQFIIWWAGLMRGAVTVALSYNAFSGSV EVSNERSLMISCTLIVVLFSTLLFGTVTKPLIRAILGAPRHAVSYASDIPSLEYLDIPFL ESQENQDNPENQETNLNGDEEPRQRRGGFSLIMKYPTGAVHHIWRKFDNKFMRPV FGGRGFVTYEPASPTGAADISHEQ
CqNHX-17	AUR62000934-RA	MWSQLGSLLSGKMDALATSDHASVVSMNLFVALLCGCIIIGHLLEENRWMNESITA LLIGLSTGVVILLISGGKSSHLLVFSEDLFFIYLLPPIIFNAGFQVKKKQFFRNFITIILF GAVGTLVSFTIISFGALSIFKKLDIGSLDLADYLAIGAIFAATDSVCTLQVLNQDETPL LYSLVFGEGVVNDATSVVLFNAIHSFDLTSIDHRIALQFMGNFLYLFIASTILGAFTG LLSAYIIKKLYFGRHSTDREVALMILMAYLSYMLAELFYLSGILTVFFCGIVMSHYT WHNVTESSRVTTKHAFATLSFVAEVFLFLYVGMDALDIEKWRFVSDSPGISVAVSSI LLGLIMVGRAAFVFPLSWLMNFAKKSQSEKVSLNQQVVIWWAGLMRGAVSMAL AYNQFTRSGHTQLRGNAIMITSTITVVLFSTMVFGLLTKPLIMFLLPQPKHFTSCSTV SDLGSPKAYSPLLEGHQDYEVDVGNHDDGTEPTIVRPSSLRMLLNAPTHTVHHYW RKFDDSFMRPVFGGRGFVPFVPGSPTEQSTHNLVDRT
CqNHX-18	AUR62015923-RA	MGESGHAQVVPISVFVAVLCFCLVIGHLLEENRWLNESITAIVIGCLTGMIILFISKGT NSHILRFDEELFFIYLLPPIIFNAGFQVKKKQFFQNFLTIMLFGVIGVFISTTIISLGSW WLLPKVGFAALTARDYLAIGTIFSSTDTVCTLQVLNQDDTPLLYSLVFGEGVVNDA TSVVLFNAIQKLNVKKVGDWTHVLGSFLYLFFASTALGIGTGLLTAYALKASYFGR HSTDREIALMTLMAYLSYTLAEASAYFCSKLRLAELSGILTVFFCGIVMSHYAWHIF ATMSFIAETFIFLYVGMDVFDMEKWKMTRLSIWTLLGIYGTIVFLILLARAAFVFPL SALSNRMNREKERSGRRSSITFKKQVIIWWAGLMRGAVSIALAFKQFTYSGVTMDT INATMVTTTVIVVLFTTLVFGFLTKPLIFYLVPHSPTQEPKSPKDDLTLPMLSMDEST ATNLSRAKDSLSMLFERPIHTIHSYWRKFDDAYMRPFFGGPSNNQRSPDSPVRNKS LFHSGFDKPALRLSPKNKG
CqNHX-19	AUR62015223-RA	MEDQLISPADVHGSPAKEQQAAGVGILLQIMMLVLSFVLGHVLRRHKFYYLPEAS ASLLIGLVVGGLANISNTETSISCVLVIVFLDSFVATQSGFILAPKPFFSNFGAIVTFSV LGTFIASLTGLLVYLGGVTYLIYKLPFVECMMFGALITSTDPITVLAIFQELGTDVNL YALVFGESVLNDAVAISLYRSMSALKGQSSSQNVFTFFLRFIETFAGSMSSVFTLVNI GLYLIYCLGGYILCNLDFMNLFKYAGLDIENLQNLECCLFVLFPYFSYMLAEGLGL

		SGIVSILFTGIVMKHYTYSNLSEKSQQFVSGFFHLISSLAETFIFIYMGLDIVMEKHS WSHIGFIFFAIITIVLARAVNVFSCGYLVNLVRPAHRKIPMTHQKALWYNGLRGAM AFALALQSVHDLPEGHGQTIFTATTAIVVLTVLLIGGTTGTMLESLQVIGDTPEGHL DGFNRSDGFVSSGQEDEGSSSNRFKMRLKEFHRSAPSFSSLDKNYLTPFFTTQTGD EEEEVPDFWAAGREEEPVLVFWKARVRRFPVLGQVRWRNSVAGAGRGWFGSWL GRRATVGGCLLVV
CqNHX-20	AUR62017800-RA	MAAYRIAVPFPLMLAEVGAETTAPMNSTISASAMAEESESNPTDAVIFFGVSLILGIA CRHFLRGTRVPYTVALLIIGIGLGSLEYGTKHGLGRFGDGIRIWENIDPELLLAVFLP ALLFESSFSMEIHQIKRCAAQMILLAGPGVLISTFCLGAALKLSFPYDWSWKTSLLL GGLLSATDPVAVVALLKELGASKKLSTIIEGESLMNDGTAIVVYQLFLKMILGRTFN WASILKYLVQVSFGAVGFGIAFGIASVLWLGFIFNDTIIEITLTLAVSYAAYFTAQEGA DVSGVLTVMTLGMFYAAAARTAFKGESQQSLHHFWEMVAYIANTLIFILSGAVIAQ GVLSSDNIFENHGTAWGYLILLYVYVLVARGVVVGVLYPFLCYFGYGMEWKEAMI LVWAGLRGAVALSLSLSVKRSSGDPAYLSTQTGTLFVFFTGGIVFLTLIINGSTTQFV LQFLGMDKLSKAKRRILEFTKYEMEKKALEAFGDLGEDEELGPADWPTVKRYIKS LNSIDGDRIHPHDASDNGFLDPMNLKDMRVRLLNGVQAAYWAMLDEGRITQSTA NVLMQSVDEALDSVDHEPLCDWKGLKNSVHFPKYYRLLQGGIYPKKLVTFFTVE RLESACYICAAFLRAHRTARGQLHDFIGYSEISSAVITESETEGEEARKFLEDVRTTF PEVLRVVKTRQVTYAVLQHLIEYIESLEKAGILEEKEMLHLHDAVQTDLKRLVRNP PTVKIPKIGELISMHPFLGALPSGVRDLLVGSTKEEVKVRGMTLYKEGGKPNGIWLI SNGVVKWACKVKKNKHALHQTVTHGSTLGLYEVLIGKPYLCDMITDSVAVCFYIE TEKILAALGSDPAVEHFFWKESVIVLAKVLLPRVFENMSMQDMRKLTAERSTLNTY LRGETIEVSSHSIGFLLEGFVKSHPLAEELIPSPAALWPAQGNSSFLSQEGSGYKSTSF LHQGTSYYVETRARVLLIDMVPIQADNTLLRRKSSLLLHDQSSRSLSSRDHAGLLS WPENQYKSYQHLPDGQEIDDSQNLSAKAMRLSIYGSTAKDVPVRGLSFQGYTLGN PSHVRSYPQVPIGQKQLPLTSVKSEGSNTVRKRLGEDVMREELLPPTHSRHPSRAV DDSSSESGGEDEVFVRIDSPSKLSFRQAP
