# Supplementary material for: Comprehensive Analysis of the NHX Gene Family and Its Regulation Under Salt and Drought Stress in Quinoa (Chenopodium quinoa Willd.)
Source: Genes (Basel). 2025 Jan 9;16(1):70. doi: 10.3390/genes16010070 (PMC11765057; doi:10.3390/genes16010070)
Supplement: Supplementary file 1 [file genes-16-00070-s001.zip › Table S3 physiochemical properties.pdf]

Table S3: Physiochemical properties of NHX genes from *Chenopodium quinoa*

Protein ID	Chromosome Location	Exons	Length (AA)	Strand	MW (kDa)	pI	GRAVY	Aliphatic Index	Instability Index	Sub-cellular Localization	TMs	No. of AA in TMs
AUR62017800-RA	Chr15: 19376789-19399189	23	1158	-	127.91	6.27	0.099	100.62	35.90	PM	12	242.35
AUR62003491-RA	Chr09: 2972519-2994911	23	1211	-	133.81	6.26	0.088	101.35	35.14	PM	12	233.94
AUR62005035-RA	Chr05: 69786235-69795161	14	551	+	60.93	6.62	0.516	107.21	33.59	Vac	12	243.27
AUR62000934-RA	Chr12: 11415996-11425350	14	551	-	60.92	6.49	0.534	109.67	34.53	Vac	12	246.16
AUR62015223-RA	Chr15: 59903188-59915082	20	581	+	63.96	5.92	0.504	106.18	49.98	Vac	10	235.86
AUR62017691-RA	Chr00: 137911025-137920676	20	514	+	57.09	5.38	0.439	103.35	50.73	Vac	9	213.81
AUR62015923-RA	Chr05: 238467-243048	14	534	+	59.86	9.18	0.439	103.71	35.37	Vac	11	244.67
AUR62024750-RA	Chr01: 46898948-46903876	14	534	-	59.96	8.06	0.525	105.86	35.78	Vac	11	236.37
AUR62000862-RA	Chr12: 10303174-10319099	14	544	-	60.14	6.08	0.470	112.52	32.40	Vac	10	233.02
AUR62005112-RA	Chr05: 70877463-70887273	14	510	+	56.30	8.97	0.586	114.86	30.46	Vac	10	234.24

Note: AA -Amino acids; MW (kDa)- Molecular weight (Kilo Daltons); pI- Isoelectric point; GRAVY- Grand average of hydrophobicity; PM-Plasma membrane; Vac- Vacuolar; TMs- Transmembrane helical domains; No. of AA in TMs- Number of amino acids in TMs
