# Supplementary material for: Comprehensive Analysis of the NHX Gene Family and Its Regulation Under Salt and Drought Stress in Quinoa (Chenopodium quinoa Willd.)
Source: Genes (Basel). 2025 Jan 9;16(1):70. doi: 10.3390/genes16010070 (PMC11765057; doi:10.3390/genes16010070)
Supplement: Supplementary file 1 [file genes-16-00070-s001.zip › Table S4 phosphorylation sites.pdf]

Table S4: The phosphorylation sites identified in *CqNHXs*

Sr. No.	Protein ID	PKC	CKII	PKA	UNSP	EGFR	INSR	PKG	CKI	DNAPK	CDC2	RSK	CDK5	GSK3	P38MAPK	ATM	SRC
1	AUR62017800-RA	35	17	19	61	4	2	4	9	7	23	5	2	2	0	3	1
2	AUR62003491-RA	38	19	23	65	4	2	2	10	7	22	4	2	3	3	2	1
3	AUR62005035-RA	20	3	10	25	1	0	3	3	1	7	1	3	3	3	1	1
4	AUR62000934-RA	19	2	11	23	1	0	3	2	2	7	1	3	3	3	1	0
5	AUR62015223-RA	17	6	4	18	0	0	0	3	5	13	0	0	1	4	2	0
6	AUR62017691-RA	15	5	1	17	0	0	0	3	4	13	1	3	1	3	1	0
7	AUR62015923-RA	16	2	9	23	1	1	2	2	2	9	2	4	4	5	1	0
8	AUR62024750-RA	17	3	7	18	1	1	1	3	2	5	1	4	0	2	1	0
9	AUR62000862-RA	19	1	9	24	0	0	1	3	2	8	1	1	1	2	3	0
10	AUR62005112-RA	18	1	10	19	0	0	1	3	0	8	1	1	1	2	2	0

Note: PKA/B/C/G: protein kinase A/B/C/G, CK I & II: casein kinase-I & II, UNSP: unspecified phosphorylation, EGFR: epidermal growth factor receptor, INSR: insulin receptor tyrosine kinase, DNAPK: DNA-dependent protein kinase, CDC2: A-type cyclin-dependent kinases, RSK: ribosomal S6 kinase, CDK5: cyclin dependent kinase 5, GSK: glycogen synthase kinase 3, p38MAPK: p38 mitogen-activated protein kinase, ATM: ataxia telangiectasia mutated and SRC: src tyrosine protein kinase.
