# Supplementary material for: Comprehensive Analysis of the NHX Gene Family and Its Regulation Under Salt and Drought Stress in Quinoa (Chenopodium quinoa Willd.)
Source: Genes (Basel). 2025 Jan 9;16(1):70. doi: 10.3390/genes16010070 (PMC11765057; doi:10.3390/genes16010070)
Supplement: Supplementary file 1 [file genes-16-00070-s001.zip › Table S5 gene divergence.pdf]

Table S5: Ka/Ks substitution ratio, duplication and selection types of NHX genes in quinoa

Duplicated gene 1	Chr Location	Duplicated gene 2	Chr Location	Type of Duplication	Ks	Ka	Ka/Ks	Selective pressure
AUR62017800-RA	Chr15	AUR62003491-RA	Chr 9	Segmental	0.082062	0.008821	0.107493	Purifying
AUR62017800-RA	Chr15	AUR62015223-RA	Chr15	Tandem	1.990670	1.036115	0.520485	Purifying
AUR62017800-RA	Chr15	AUR62017691-RA	Chr0	Segmental	1.886835	1.051054	0.557046	Purifying
AUR62003491-RA	Chr9	AUR62015223-RA	Chr15	Segmental	1.871077	1.025829	0.548256	Purifying
AUR62003491-RA	Chr9	AUR62017691-RA	Chr0	Segmental	1.739176	1.046073	0.601476	Purifying
AUR62003491-RA	Chr9	AUR62000862-RA	Chr12	Segmental	2.976076	1.046224	0.351544	Purifying
AUR62003491-RA	Chr9	AUR62005112-RA	Chr5	Segmental	2.662007	1.047052	0.393331	Purifying
AUR62005035-RA	Chr5	AUR62000934-RA	Chr12	Segmental	0.122385	0.007065	0.057729	Purifying
AUR62005035-RA	Chr5	AUR62015223-RA	Chr15	Segmental	3.200221	0.873996	0.273105	Purifying
AUR62005035-RA	Chr5	AUR62000862-RA	Chr12	Segmental	2.139333	0.300655	0.140536	Purifying
AUR62005035-RA	Chr5	AUR62005112-RA	Chr5	Tandem	2.125749	0.300788	0.141497	Purifying
AUR62000934-RA	Chr12	AUR62000862-RA	Chr12	Tandem	2.071634	0.299511	0.144577	Purifying
AUR62000934-RA	Chr12	AUR62005112-RA	Chr5	Segmental	2.171989	0.299644	0.137958	Purifying
AUR62015223-RA	Chr15	AUR62017691-RA	Chr0	Segmental	0.066504	0.028624	0.430407	Purifying
AUR62015223-RA	Chr15	AUR62015923-RA	Chr5	Segmental	2.309710	0.836822	0.362306	Purifying
AUR62015223-RA	Chr15	AUR62024750-RA	Chr1	Segmental	2.383748	0.829549	0.348001	Purifying
AUR62015223-RA	Chr15	AUR62000862-RA	Chr12	Segmental	2.763261	0.944552	0.341825	Purifying
AUR62015223-RA	Chr15	AUR62005112-RA	Chr5	Segmental	2.731599	0.941418	0.344639	Purifying
AUR62017691-RA	Chr0	AUR62015923-RA	Chr5	Segmental	2.466885	0.830498	0.336658	Purifying
AUR62017691-RA	Chr0	AUR62000862-RA	Chr12	Segmental	2.499501	0.949965	0.380062	Purifying
AUR62017691-RA	Chr0	AUR62005112-RA	Chr5	Segmental	2.684934	0.946810	0.352638	Purifying
AUR62015923-RA	Chr5	AUR62024750-RA	Chr1	Segmental	0.177050	0.045653	0.257856	Purifying
AUR62000862-RA	Chr12	AUR62005112-RA	Chr5	Segmental	0.100208	0.005439	0.054284	Purifying

Note: Chr: Chromosomal location; Ka: non-synonymous substitution; Ks: synonymous substitution.
